# Distal interphalangeal joint involvement in patients with rheumatoid arthritis: Where are we?

**DOI:** 10.55730/1300-0144.5847

**Published:** 2024-03-23

**Authors:** Mete PEKDİKER, Sertaç KETENCİ, Gökhan SARGIN

**Affiliations:** 1 Department of Rheumatology-Internal Medicine, Faculty of Medicine, Hatay Mustafa Kemal University, Hatay, Turkiye; 2 Department of Rheumatology-Physical Therapy and Rehabilitation, Faculty of Medicine, Samsun Ondokuz Mayıs University, Samsun, Turkiye; 3 Department of Rheumatology-Internal Medicine, Faculty of Medicine, Aydın Adnan Menderes University, Aydın, Turkiye

**Keywords:** Distal interphalangeal joint, erosive arthritis, rheumatoid arthritis

## Abstract

**Background/aim:**

Rheumatoid arthritis (RA) usually affects the wrist, metacarpophalangeal joint, and proximal interphalangeal joint of the hands. However, the distal interphalangeal (DIP) joints may also be involved in RA patients. In this study, we aimed to evaluate the frequency and associated factors of DIP joint erosion in patients with RA.

**Materials and methods:**

Medical records of patients with RA were reviewed retrospectively. Patients with major trauma affecting DIP joints, osteoarthritis, erosive osteoarthritis, psoriatic arthritis, systemic sclerosis, calcium pyrophosphate dihydrate disease, and gout were excluded. Anteroposterior hand X-rays were evaluated and patients were divided into groups according to autoantibody profile.

**Results:**

We reviewed 1213 patients with a mean age of 54.3 ± 12.5 years; 82.8% of them were female, and 95.4% had RA-type erosive changes. The DIP erosion rate was 12%. DIP involvement was generally unilateral and asymmetric, with the 3rd finger being the most commonly affected joint. Patients with DIP erosions had a significantly longer disease duration (p = 0.036). Older age was an independent predictive factor for DIP erosion (p = 0.001).

**Conclusion:**

In this large-sample study, we reported DIP joint involvement in patients with RA. Advanced age could have affected the results because hand erosions increase above 50 years in a healthy population. Our results may provide a different perspective on joint involvement in RA.

## 1. Introduction

Rheumatoid arthritis (RA) is a chronic, multisystemic, and autoimmune rheumatic disease that affects synovial joints and causes joint erosions. It is one of the most common inflammatory rheumatic diseases. The disease decreases work capacity and quality of life during RA [1]. Joint erosion occurs in 90% of patients with RA [2]. The 1987 American College of Rheumatology (ACR) RA revised classification criteria including clinical and radiological evaluation of hand joints such as metacarpophalangeal (MCP), and proximal interphalangeal (PIP) joints [3]. The 2010 ACR/European League against Rheumatism (EULAR) RA classification criteria include clinical assessment of hand joints (wrist, MCP, and PIP) [4]. Distal interphalangeal (DIP) joint involvement does not receive any points in the current classification criteria. Disease activity score-28 (DAS-28), which is the most commonly used RA disease activity scoring system, does not include DIP joint arthritis [5]. Modified Sharp score (mSS) is one of the radiographic scoring systems evaluating joint damage in patients with RA, and it includes 15 areas for joint space narrowing (JSN) and 16 areas for bone erosion, but neither erosion score nor JSN score includes DIP joints [6]. Distal interphalangeal joint involvement with radiological changes is well-defined in rheumatic diseases such as erosive osteoarthritis (EOA), hand osteoarthritis (OA), and psoriatic arthritis (PsA) [7,8]. Adult-onset Still’s disease, anti-Jo-1 syndrome, calcium pyrophosphate dihydrate disease (CPPD), multicentric reticulohistiocytosis, and gout can also affect the DIP joints [9–13].

In our daily rheumatology practice, we encounter arthritis and/or arthralgia in DIP joints in patients with RA. All diseases that may cause DIP involvement are evaluated. In this large sample-sized study, we aimed to find the frequency and associated factors of DIP joint erosions in patients with RA.

## 2. Materials and methods

We evaluated the RA patients followed up in our rheumatology department, who were 18 years of age or older, and who had an anterior-posterior hand X-ray within the last year were included in the study. The ACR/EULAR 2010 RA classification criteria were used to diagnose RA [4]. Demographic, laboratory, clinical, and treatment characteristics were noted from electronic medical records, retrospectively. The exclusion criteria included patients who had lost a large portion of the finger (including at least one DIP joint), had a history of fractures in the hand bones, or had overlapping rheumatologic or non-rheumatologic diseases that can cause erosions or deformities in the DIP joints, such as systemic sclerosis (SSc), PsA, CPPD, gout, EOA, and hand osteoarthritis (OA). Patients having hand deformities or signs on hand X-rays that were well defined for EOA or PsA, such as saw-tooth, gull-wing, mouse-ear, terminal tuft erosion, acro-osteolysis, or fluffy periostitis, were also excluded [8,14].

Diagnosis of joint erosion in DIP joints was based on EULAR definition as ‘interruption of the cortex of the bone’ [15]. ‘Rheumatoid arthritis type joint involvement (RJI)’ was based on mSS [6], and defined as having any joint erosion or JSN. ‘Serious joint involvement (SJI)’ was defined as having any erosion score ≥3 points or JSN score ≥4 points according to mSS. All of the hand X-rays were evaluated separately by the rheumatologists who were blinded to the patients. If there was no agreement between readers, X-rays were reevaluated by all readers, then a final common decision was made with full agreement. A nephelometric assay detected rheumatoid factor (RF); serum samples with results ≥14 IU/mL were defined as positive. Anticyclic citrullinated peptide antibody-2 IgG (anti-CCP) was detected by enzyme-linked immunosorbent assay; serum samples with results ≥5 U/mL were defined as positive. The study was approved by the local Ethics Committee and was conducted following the principles of the 1964 Declaration of Helsinki and its later amendments (approval no: 2023/03).

Statistical analysis was performed using SPSS 22.0 version (IBM SPSS, Chicago, IL). The results were given as a number, frequency, mean ± standard deviation, and/or median [25–75p] value. The chi-squared test and Fisher’s exact test were used for the analysis of categorical data and independence between variables. The Mann–Whitney U test and independent-samples t-test were used to compare differences between groups according to the distribution analyses. Logistic regression analysis was performed to calculate the estimated values of the dependent variable as probabilities and to classify according to probability rules. The results were assessed at a 95% confidence interval, and a p-value of less than 0.05 was accepted as significant.

## 3. Results

We reviewed 1213 patients with a mean age of 54.3 ± 12.5 years, and 82.8% of them were female. The median disease duration was 5 [2–11] years. The rate of smoking history (active or ex) was 31.8%. Rheumatoid arthritis type joint involvement and SJI were observed in 95.4% and 24.7% of patients, respectively. We found the rate of DIP joint erosion as 12%. All of the patients with DIP erosions had a positive history of tenderness and swelling on DIP joints. None of the patients with DIP erosions had a positive family history (in first- and second-degree relatives) for PsA.

The minimum and maximum numbers of eroded DIP joints in patients were one and four, respectively. Only nine patients (6.1%) had bilateral-symmetrical DIP joint erosions. Isolated DIP joint erosion was absent. The most commonly affected DIP joint was the 3rd finger. Demographic, laboratory, clinical, and treatment characteristics are presented in Table 1. Table 2 shows the general characteristics of patients with and without DIP involvement. Both the RF- and anti-CCP–negative groups had a DIP erosion rate of 13.1%. When evaluated according to the autoantibody profile, there were no significant differences between all groups (Table 3).

In multivariate analysis, age was the independent predictive factor for DIP joint erosions (p = 0.001). Disease duration was the predictive factor for DIP erosion (p = 0.036), and there was no relationship between DIP joint erosion, sex, smoking, RF, anti-CCP, RJI, SJI, and biological agent use (p > 0.05).

## 4. Discussion

In this large-sample study, we evaluated the frequency and associated factors of DIP joint erosion in patients with RA. We found that erosive DIP joint involvement was 12.0%. The most commonly affected finger was the 3rd DIP. Distal interphalangeal joint erosions generally exhibited a unilateral-asymmetric pattern. Age emerged as an independent predictive factor for DIP joint erosions (p = 0.001).

Jacob et al. reported a higher rate of DIP joint erosion in seropositive RA patients compared to the age- and sex-matched control group (37% versus 14%) [16]. In their study, isolated DIP joint involvement was absent, the most commonly affected joint was the 3rd DIP, and DIP joint involvement was generally unilateral [16]. These findings are consistent with those of our study. In another study, the rate of DIP joint erosion was 16% in patients with RA. Halla et al. reported that the 2nd and 5th DIPs were the most commonly affected joints [17]. In addition, they reported the predominance of asymmetric patterns and the absence of isolated DIP involvement in RA patients [17]. Papasavvas et al. reported the rate of DIP joint erosion as 12% in patients with RA, with 70% of DIP joint erosion presenting an asymmetrical pattern [18]. In a prospective study, erosive changes in DIP joints were 5.3% at the disease onset and 14.9% in the following third years in patients with RA [19]. The exclusion of OA is a cornerstone in the studies involving hand articulations. Because RA patients have an increased risk of developing OA than the non-RA population and OA is associated with enhanced marginal erosions in DIP joints in patients with RA [20,21].

In one study, DIP joint erosion was present in 12% of the RA group which was nearly half of the prevalence seen in the PsA group [22]. Another study reported a significantly higher rate of DIP erosion in patients with PsA compared to those with RA [23]. In this study, the mean age of RA patients was similar, but the mean disease duration was shorter compared to our study results [23]. In our study, both seropositive and seronegative RA patients had a DIP joint erosion rate of 11.5% and 12.7%, respectively (p > 0.05). Ikemura et al. identified an association between DIP joint erosion and advanced age, long disease duration, and PIP joint erosion [24]. Mizuuchi et al. reported a clinical DIP joint involvement rate of 2.1% in RA without any radiological evidence. Patients with clinical DIP involvement were significantly younger, and female patients were more frequently affected [25].

Limitations of our study included its retrospective nature, intra- and interobserver differences, lack of total modified Sharp score (mSS), and absence of imaging evidence such as ultrasonography or contrast-enhanced magnetic resonance imaging to detect synovitis. Distal interphalangeal joint synovitis can be documented by ultrasonography and indocyanine green-enhanced fluorescence optical imaging in patients with RA [26].

In conclusion, we detected DIP joint erosion in %12 of RA patients and identified age as an independent predictive factor for developing DIP joint erosion in RA. Advanced age could have affected our results because hand erosions increase above 50 years in a healthy population [27]. Our results may provide insights into the consideration of DIP involvement in RA patients and its evaluation and differential diagnosis.

## Figures and Tables

**Table 1:** The demographic, laboratory, clinical, and treatment characteristics.

Total patient count, n	1213

Male %, (n)	17.2 (209)

Female %, (n)	82.8 (1004)

Smoking history %, (n)	31.8 (386)

Age (mean standard deviation, years)	54.3 ± 12.5

Disease duration time (median [25–75p], years)	5 [2–11]

Rheumatoid factor positivity %, (n)	56.6 (686)

Anti-CCP positivity %, (n)	54.0 (655)

Biologic agent use %, (n)	30.8 (373)

Rheumatoid arthritis type joint involvement %, (n)	95.4 (1157)

Serious joint involvement %, (n)	24.7 (300)

Patients with DIP erosion %, (n)	12.0 (146)

Distribution of DIP erosions %, (n)	
□ 2.DIP	□ 12.5% (24)
□ 3.DIP	□ 41.9% (80)
□ 4.DIP	□ 26.7% (51)
□ 5.DIP	□ 18.9% (36)

Abbreviations: DIP, distal interphalangeal; anti-CCP, anticyclic citrullinated peptide.

**Table 2:** The general characteristics of patients with and without DIP involvement.

Variable	DIP joint involvement (−) group	DIP joint involvement (+) group
Total patient count, n	1067	146
Male %, (n)	18.1 (193)	11 (16)
Female %, (n)	81.9 (874)	89 (130)
Smoking history %, (n)	32.1 (343)	29.5 (43)
Age (mean ± standard deviation, years)	53.9±12.5	57.6±12.2
Disease duration time (median [25–75p], years)	5 [2–11]	6 [2–12]
RF positivity %, (n)	56.8 (606)	54.8 (80)
Anti-CCP positivity %, (n)	54.2 (579)	52 (76)
Biologic agent history %, (n)	30.3 (324)	33.5 (49)
Rheumatoid arthritis type joint involvement %, (n)	95 (1014)	98 (143)
Serious joint involvement %, (n)	24.2 (259)	28 (41)

Abbreviations: DIP, distal interphalangeal; RF, rheumatoid factor anti-CCP, anticyclic citrullinated peptide.

**Table 3:** Classification of patients according to autoantibodies.

Patient groups	Patient with DIP erosion/total patient, n, (%)
Group 1: RF (+) and Anti-CCP (+)	67/570 (11.8)
Group 2: RF (+) and Anti-CCP (−)	12/116 (10.3)
Group 3: RF (−) and Anti-CCP (+)	9/85 (10.6)
Group 4: RF (−) and Anti-CCP (−)	58/442 (13.1)

Abbreviations: DIP, distal interphalangeal; RF, rheumatoid factor; anti-CCP, anticyclic citrullinated peptide.

## References

[1] Smolen JS Aletaha D Barton A Burmester GR Emery P Rheumatoid arthritis Nature Review Disease Primers 2018 4 18001 10.1038/nrdp.2018.1 29417936

[2] Fleming A Benn R Corbett M Wood P Early rheumatoid disease II. Patterns of joint involvement Annals of Rheumatic Disease 1976 35 4 361 364 10.1136/ard.35.4.361 PMC1007397 970995

[3] Arnett FC Edworthy SM Bloch DA McShane DJ Fries JF The American Rheumatism Association 1987 revised criteria for the classification of rheumatoid arthritis Arthritis and Rheumatism 1988 31 3 315 324 10.1002/art.1780310302 3358796

[4] Neogi T Aletaha D Silman AJ Naden RL Felson DT The 2010 American College of Rheumatology/European League against Rheumatism classification criteria for rheumatoid arthritis: Phase 2 methodological report Arthritis and Rheumatism 2010 62 9 2582 2591 10.1002/art.27580 20872596 PMC3077961

[5] van Gestel AM Haagsma CJ van Riel PL Validation of rheumatoid arthritis improvement criteria that include simplified joint counts Arthritis and Rheumatism 1998 41 10 1845 1850 10.1002/1529-0131(199810)41:10<1845::AID-ART17>3.0.CO;2-K 9778226

[6] Sharp JT Young DY Bluhm GB Brook A Brower AC How many joints in the hands and wrists should be included in a score of radiologic abnormalities used to assess rheumatoid arthritis? Arthritis and Rheumatism 1985 28 12 1326 1335 10.1002/art.1780281203 4084327

[7] Zhang W Doherty M Leeb BF Alekseeva L Arden NK EULAR evidence-based recommendations for the diagnosis of hand osteoarthritis: report of a task force of ESCISIT Annals of the Rheumatic Diseases 2009 68 1 8 17 10.1136/ard.2007.084772 18250111

[8] Shiraishi M Fukuda T Igarashi T Tokashiki T Kayama R Differentiating Rheumatoid and Psoriatic Arthritis of the Hand: Multimodality Imaging Characteristics Radiographics 2020 40 5 1339 1354 10.1148/rg.2020200029 32735474

[9] Belghali S El Amri N Baccouche K Laataoui S Bouzaoueche M Atypical form of adult-onset Still’s disease with distal interphalangeal joints involvement Current Rheumatology Reviews 2018 14 3 284 288 10.2174/1573397113666170728124845 28758587

[10] Kumar RR Jha S Dhooria A Naidu GSRSNK Minz RW Anti–Jo-1 syndrome often misdiagnosed as rheumatoid arthritis (for many years): a single-center experience Journal of Clinical Rheumatology 2021 27 4 150 155 10.1097/RHU.0000000000001234 31895110

[11] Dieppe PA Alexander GJ Jones HE Doherty M Scott DG Pyrophosphate arthropathy: a clinical and radiological study of 105 cases Annals of the Rheumatic Diseases 1982 41 4 371 376 10.1136/ard.41.4.371 7114920 PMC1000953

[12] Santilli D Lo Monaco A Cavazzini PL Trotta F Multicentric reticulohistiocytosis: a rare cause of erosive arthropathy of the distal interphalangeal finger joints Annals of the Rheumatic Diseases 2002 61 6 485 487 10.1136/ard.61.6.485 12006317 PMC1754120

[13] Hegaze AH Hamdi AS Alqrache A Hegazy M Unusual case of gouty arthritis of the second distal interphalangeal joint (second toe) Cureus 2020 12 11 e11405 10.7759/cureus.11405 33312803 PMC7725467

[14] Punzi L Ramonda R Sfriso P Erosive osteoarthritis Best Practice & Research Clinical Rheumatology 2004 18 5 739 758 10.1016/j.berh.2004.05.010 15454130

[15] Knevel R Lukas C van der Heijde D Rincheval N Combe B Defining erosive disease typical of RA in the light of the ACR/EULAR 2010 criteria for rheumatoid arthritis; results of the data driven phase Annals of the Rheumatic Diseases 2013 72 4 590 595 10.1136/annrheumdis-2012-202778 23393145

[16] Jacob J Sartoris D Kursunoglu S Pate D Pineda CJ Distal interphalangeal joint involvement in rheumatoid arthritis Arthritis and Rheumatism 1986 29 1 10 15 10.1002/art.1780290102 3947406

[17] Halla JT Fallahi S Hardin JG Small joint involvement: a systematic roentgenographic study in rheumatoid arthritis Annals of the Rheumatic Diseases 1986 45 4 327 330 10.1136/ard.45.4.327 3707221 PMC1001878

[18] Papasavvas GK Thompson PW Kirwan JR Small joint involvement: systematic roentgenographic study in rheumatoid arthritis Annals of the Rheumatic Diseases 1987 46 4 351 352 10.1136/ard.46.4.351-b PMC1002138 3592797

[19] Brook A Corbett M Radiographic changes in early rheumatoid disease Annals of the Rheumatic Diseases 1977 36 1 71 73 10.1136/ard.36.1.71 843114 PMC1006633

[20] Lee YH Tsou HK Kao SL Gau SY Bai YC Patients with rheumatoid arthritis increased risk of developing osteoarthritis: a nationwide population-based cohort study in Taiwan Frontiers of Medicine (Lausanne) 2020 7 392 10.3389/fmed.2020.00392 PMC7511507 33015077

[21] Abbott GT Bucknall RC Whitehouse GH Osteoarthritis associated with distal interphalangeal joint involvement in rheumatoid arthritis Skeletal Radiology 1991 20 7 495 497 10.1007/BF00194244 1754910

[22] Wright V Psoriatic arthritis: a comparative radiographic study of rheumatoid arthritis and arthritis associated with psoriasis Annals of the Rheumatic Diseases 1961 20 2 123 132 10.1136/ard.20.2.123 13786830 PMC1007195

[23] Ichikawa N Taniguchi A Kobayashi S Yamanaka H Performance of hands and feet radiographs in differentiation of psoriatic arthritis from rheumatoid arthritis International Journal of Rheumatic Diseases 2012 15 5 462 467 10.1111/j.1756-185X.2012.01818.x 23083036

[24] Ikemura S Hagio S Akasaki Y Fujiwara T Tsushima H Frequency and risk factor analyses of bone erosion of the distal interphalangeal joint in patients with rheumatoid arthritis: a cross-sectional study Acta Reumatológica Portuguesa 2021 46 3 239 245 34628456

[25] Mizuuchi T Sawada T Nishiyama S Tahara K Hayashi H Distal interphalangeal joint involvement may be associated with disease activity and affected joint distribution in rheumatoid arthritis Journal of Clinical Medicine 2022 11 5 1405 10.3390/jcm11051405 35268496 PMC8911492

[26] Glimm AM Werner SG Burmester GR Backhaus M Ohrndorf S Analysis of distribution and severity of inflammation in patients with osteoarthitis compared to rheumatoid arthritis by ICG-enhanced fluorescence optical imaging and musculoskeletal ultrasound: a pilot study Annals of the Rheumatic Diseases 2016 75 3 566 570 10.1136/annrheumdis-2015-207345 26311723 PMC4789689

[27] Berlin A Simon D Tascilar K Figueiredo C Bayat S The ageing joint-standard age- and sex-related values of bone erosions and osteophytes in the hand joints of healthy individuals Osteoarthritis and Cartilage 2019 27 7 1043 1047 10.1016/j.joca.2019.01.019 30890457

